# Diverse functionality among human NK cell receptors for the C1 epitope of HLA-C: KIR2DS2, KIR2DL2, and KIR2DL3

**DOI:** 10.3389/fimmu.2012.00336

**Published:** 2012-11-22

**Authors:** Achim K. Moesta, Peter Parham

**Affiliations:** ^1^Genome Analysis Unit, Discovery Research, Amgen Inc., South San Francisco, CA, USA; ^2^Department of Structural Biology, Stanford UniversityStanford, CA, USA

**Keywords:** killer cells, natural, killer cell immunoglobulin-like receptor, receptor–ligand interaction, disease association, structure–function relationship

## Abstract

Interactions between killer immunoglobulin-like receptors (KIRs) and their HLA-A, -B, and -C ligands diversify the functions of human natural killer cells. Consequently, combinations of KIR and HLA genotypes affect resistance to infection and autoimmunity, success of reproduction and outcome of hematopoietic cell transplantation. HLA-C, with its C1 and C2 epitopes, evolved in hominids to be specialized KIR ligands. The system’s foundation was the C1 epitope, with C2 a later addition, by several million years. The human inhibitory receptor for C1 is encoded by *KIR2DL2/3*, a gene having two divergent allelic lineages: *KIR2DL2* is a *B KIR* haplotype component and *KIR2DL3* an *A KIR* haplotype component. Although KIR2DL2 and KIR2DL3 exhibit quantitative differences in specificity and avidity for HLA-C, they qualitatively differ in their genetics, functional effect, and clinical influence. This is due to linkage disequilibrium between KIR2DL2 and KIR2DS2, a closely related activating receptor that was selected for lost recognition of HLA-C.

Natural killer (NK) cells contribute to immune defense against infection (Biron et al., 1999; Orange, 2002), and also to forming the placenta during reproduction (Hiby et al., 2004). In order to kill infected cells selectively, NK cells must distinguish healthy cells from diseased cells and cause them no harm (Ljunggren and Karre, 1990). A mechanism for achieving this, involves inhibitory NK cell receptors that engage MHC class I molecules. By their level of expression and diverse repertoire of bound peptides, MHC class I molecules provide NK cell receptors with a sensitive read-out of cellular health. Humans have two complementary types of inhibitory receptor that recognize human MHC (HLA) class I molecules. Recognition of HLA-E by CD94:NKG2A involves both a highly conserved ligand and a highly conserved receptor, and is thus a constant feature of human immune systems (Borrego et al., 1998; Braud et al., 1998; Lee et al., 1998). At the opposite end of the spectrum are the extraordinarily diverse interactions between polymorphic HLA-A, -B, and -C molecules and the family of variable killer immunoglobulin-like receptors (KIR), interactions that diversify and individualize human immune systems (Valiante et al., 1997; Vilches and Parham, 2002). Although different KIR recognize HLA-A, -B, and -C, it is the interactions between KIR and HLA-C that dominate in preventing NK cells from attacking healthy autologous cells (Colonna et al., 1993; Valiante et al., 1997).

## KIR STRUCTURE, FUNCTION, AND SPECIFICITY FOR HLA CLASS I

In the context of KIR recognition of HLA-C, two mutually exclusive groups of HLA-C allotypes are defined and correlated with the sequence dimorphism at position 80 in the α_1_ domain (Colonna et al., 1993; Moretta et al., 1993). Roughly half of the >1000 known HLA-C allotypes have the asparagine at position 80 that forms the C1 epitope. The remaining HLA-C allotypes have the lysine at position 80 that forms the C2 epitope. Recognition of C1 is mediated by the inhibitory receptors, KIR2DL2 and KIR2DL3, both of which have lysine at position 44. Genomic characterization and population studies of the *KIR* gene family show that the genes encoding *KIR2DL2* and *KIR2DL3* segregate as alleles of a single genetic locus, which is often referred to as *KIR2DL2/3* (Uhrberg et al., 2002; Robinson et al., 2010, 2011). The inhibitory receptor that principally recognizes the C2 epitope of HLA-C is KIR2DL1, with some additional contribution from KIR2DL2, and possibly also KIR2DL3. In addition to the inhibitory receptors, KIR2DL1 and KIR2DL2/3 have short-tailed, activating counterparts, KIR2DS1 and KIR2DS2, respectively, whose extracellular Ig-like domains are highly homologous to those of their inhibitory partners.

Phylogenetic and structural analyses show how the KIR of humans and great apes (the hominid species) group into four discrete lineages: I, II, III, and V. All KIR that recognize HLA-C, and its orthologs in other hominid species, are of the lineage III KIR. In humans, lineage III KIR are characterized by having two extracellular Ig-like domains, D1 and D2, encoded by exons 4 and 5, respectively. Exons 1 and 2 encode the leader peptide, whereas exon 3, which encodes an Ig-like domain, is not incorporated into mRNA and is called pseudoexon 3 (Vilches et al., 2000). The presence of the pseudoexon 3 argues that the lineage III KIR with two Ig-like domains likely evolved from lineage III KIR with three Ig-like domains (D0, D1, and D2). Consistent with this thesis, some of the chimpanzee lineage III KIR that recognize Patr-C, the chimpanzee ortholog of HLA-C, have a pseudoexon 3, whereas the others have a functional exon 3 that specifies the D0 domains of these three Ig-like domains (Khakoo et al., 2000; Moesta et al., 2009).

Interaction of KIR with HLA class I is sensitive to single amino acid substitutions in the KIR, which can change its epitope specificity (Winter and Long, 1997) or eliminate recognition of HLA-C (Biassoni et al., 1997; Winter and Long, 1997; Winter et al., 1998). Winter and Long (1997) demonstrated that in the context of KIR2DL1 and KIR2DL3, mutagenesis at position 44 was sufficient to “swap” the C1 and C2 specificities. Thus the KIR2DL1 mutant with lysine-44 acquired C1 specificity, whereas the KIR2DL3 mutant with methionine 44 acquired C2 specificity. On this basis, position 44 has been described as the specificity-determining position of the lineage III KIR (Vilches and Parham, 2002). In contrast, at the adjacent position, replacement of phenylalanine 45 in KIR2DL2 with tyrosine abrogates all interaction with HLA-C. Tyrosine 45 occurs naturally in KIR2DS2, the activating counterpart of KIR2DL2/3. The sequences of the D1 and D2 domains of KIR2DS2 are very similar to those of KIR2DL2/3, particularly KIR2DL2. Despite the overall similarity, the presence of tyrosine 45 prevents KIR2DS2 from recognizing HLA-C; substituting phenylalanine for tyrosine at position 45 is sufficient for KIR2DS2 to recognize the C1 epitope (Saulquin et al., 2003). Similar swap mutagenesis between inhibitory KIR2DL1 and activating KIR2DS1 demonstrated that lysine-70, naturally present in KIR2DS1, reduced affinity for HLA-Cw4 to about half that achieved with threonine-70, naturally present in KIR2DL1 (Biassoni et al., 1997; Moesta et al., 2010; Hilton et al., 2012).

In contrast to KIR2DS2, interaction of KIR2DS1 with C2 has been well established using both cell-based assays of immunological function and direct biochemical measurements of binding (see **Table 1**). Both types of assay show that the potency of KIR2DS1 binding to C1 is significantly reduced compared to that of KIR2DL1. Functional interactions between KIR2DS1 and C2 can also be inferred from the protection that maternal KIR2DS1 affords against pregnancy disorders, such as preeclampsia and recurrent miscarriage (Hiby et al., 2010), as well as the impact of KIR2DS1 on NK cell education (Fauriat et al., 2010). Although KIR2DS2 can deliver activating signals to NK cells, the function of this enigmatic receptor remains unresolved, because a variety of experimental approaches have detected either very weak interaction with HLA class I or no interaction at all (see **Table 1**). For example, Moesta et al. (2010) observed a low but significant binding to C1^+^ HLA-C*16:01 but not to six other C1^+^ HLA-C allotypes or any HLA-A or -B allotypes. Stewart et al. (2005) demonstrated a very low binding of KIR2DS2 tetramers to EBV-infected B cells derived from donors bearing C1, but not to primary B cells or EBV-infected B cells from C2 homozygous donors, leaving open the possibility that KIR2DS2 functions under inflammatory conditions.

**Table 1:** Direct HLA interactions of KIR2DS1 and KIR2DS2.

Assay	HLA class I ligand	Reference
**KIR2DS1**		
Binding of 2DS1-Fc to HLA class I transfected 721.221 cells	HLA-C*06	Pende et al. (2009)
Recognition and killing of C2/C2 leukemic blast cells	HLA-C*04/*05 and HLA-C*04/06	Pende et al. (2009)
Binding of tetramers to C2^+^ HLA-C loaded with specific peptides	HLA-C*04:01 and C*06:02 2DL1 ≫ 2DS1	Stewart et al. (2005)
Binding of 2DS1-Fc to-bead bound HLA class I	All seven C2 tested	Moesta et al. (2010)
Induced cytotoxicity and IFN-g release against C2-bearing target cells	Not identified	Chewning et al. (2007)
2DS1-mediated killing against C2-bearing PHA blast cells	C1/C2 or C2/C2 PHA blasts	Foley et al. (2008)
**KIR2DS2**
Failure to bind HLA-C tetramers	HLA-C*03:04	Saulquin et al. (2003)
Failure of 2DS2-Fc to bind HLA class I transfected 721.221	HLA-C*01:02, -C*03:04, -C*07:02	Winter et al. (1998)
Failure of 2DS2 to bind C1 in surface plasmon resonance analysis	HLA-C*07	Vales-Gomez et al. (1998)
Weak but detectable binding of 2DS2 tetramers to C1 with particular peptides	HLA-C*03:02	Stewart et al. (2005)
Weak binding of 2DS2-Fc to a single bead-bound C1 HLA-C allotype	HLA-C*16:01	Moesta et al. (2010)

The crystal structures of several KIR alone and of two complexes of KIR bound to HLA-C (**Table 2**), provide insight to the molecular interactions that govern KIR interactions with HLA class I and determine the C1 and C2 specificities (Boyington et al., 2000; Fan et al., 2001). Whereas lysine-44 of KIR2DL2 forms a direct hydrogen bond with C1-determining asparagine-80 of HLA-C*03 (Boyington et al., 2000), the KIR2DL1/HLA-C*04 structure revealed no direct interaction between methionine 44 of KIR2DL1 and lysine-80 of HLA-C*04 (Fan et al., 2001). Instead, rather than interacting directly with HLA-C, methionine-44 contributes spatially to a charged pocket of KIR2DL1 that accommodates the lysine-80 of HLA-C*04. Biochemically, the KIR–MHC interactions are characterized by fast association and dissociation rates, with an overall affinity in the low micromolar range (Maenaka et al., 1999a). The majority of the direct interactions between KIR2DL and HLA-C are achieved by shape and charge complementarity between the two contacting surfaces. The importance of charge complementarity, can explain how single amino acid substitutions can have such profound effects as altering the specificity of the interaction or reducing its avidity to a non-detectable level.

**Table 2:** Crystal structures of HLA-C reactive KIR.

Structure	Reference	Hinge angle	PDB accession #
**Free structures**
KIR2DL1	Fan et al. (1997)	55	1NKR
KIR2DL2	Snyder et al. (1999)	84	2DLI, 2DL2
KIR2DL3	Maenaka et al. (1999b)	78	1B6U
KIR2DS2	Saulquin et al. (2003)	73	1M4K
KIR2DS4	Graef et al. (2009)	69	3H8N
**Complex structures**
KIR2DL1/C*04:01	Fan et al. (2001)	66	1IM9
KIR2DL2/C*03:04	Boyington et al. (2000)	81	1EFX botrule

Binding and functional studies show that KIR2DL2 is a stronger inhibitory receptor than KIR2DL3 (Winter et al., 1998; Moesta et al., 2008), even though the four extracellular substitutions (at positions 16, 35, 148, and 200) that distinguish the two receptors are located away from the binding site. The paired polymorphisms at residues 16 and 148 act synergistically, in a way that might control the flexibility and/or the angle of the hinge between the D1 and D2 domains (Moesta et al., 2008). The observed hinge angle in KIR crystal structures is unusually acute compared to other hematopoietic Ig-superfamily receptors (Fan et al., 1997). It is also found to vary in the structures of KIR alone, compared to the structures of KIR bound to HLA-C (Fan et al., 2001; Boyington et al., 2001). Flexibility of the hinge may, therefore, be critical for enhancing the interaction of KIR2DL2 with HLA-C.

Taken together, these studies suggest that instead of a binary “on–off” switch, KIR-mediated NK cell control involves a continuum along which the strengths of the inhibitory KIR/HLA-C interactions vary (Winter et al., 1998; Moesta et al., 2008). Among these, KIR2DL1 interaction with C2 is considered the strongest inhibitory combination, with KIR2DL2/C1 conferring intermediate inhibition, and KIR2DL3/C1 having the weakest inhibitory effect. These differences in the observed inhibitory capacities of HLA-C reactive KIR are hypothesized to explain clinical associations with the progress of viral infection outcome and reproductive success. In modulating the resolution of acute Hepatitis C virus (HCV) infection, the weaker inhibition conferred by KIR2DL3/C1 was found to be protective, possibly because it facilitates stronger NK cell responses than KIR2DL2/C1 or KIR2DL1/C2 (Khakoo et al., 2004). By contrast, in pregnancy the stronger inhibitory interaction of maternal KIR2DL1 with fetal C2 appears to render uterine NK cells hypofunctional, thereby predisposing pregnancies with this genetic combination to several types of disorders: recurrent miscarriage, preeclampsia, and fetal growth restriction (Hiby et al., 2004, 2010).

KIR2DL1 appears exquisitely specific for C2, exhibiting no detectable cross-reactivity with C1 (Moesta et al., 2008). Conversely, KIR2DL2, and to lesser extent KIR2DL3, cross-reacts with C2 and may use it as a functional ligand (see **Table 3**). C2 binds soluble KIR2DL2 in cell-free assays (Winter et al., 1998; Moesta et al., 2008), and can inhibit KIR2DL2 expressing NK cell lines (Winter et al., 1998; Moesta et al., 2008; Schonberg et al., 2011) as well as KIR2DL2^+^ NK cells (Pende et al., 2009; Schonberg et al., 2011). This interaction, of C2 with KIR2DL2 is weaker than that between C2 and KIR2DL1, but significantly stronger than that of KIR2DL3 with C2. Pointing to the physiological importance of the C2–KIR2DL2 interaction, analysis of KIR repertoire formation demonstrates that C2 can function as a ligand for KIR2DL2 *in vivo*. Notably the presence of *KIR2DL2* reduces the frequency of NK cells expressing KIR2DL1, regardless of HLA-C genotype (Schonberg et al., 2011). This effect on receptor acquisition was not apparent for KIR2DL3, suggesting its C2 reactivity is too weak *in vivo*. Because, genetically, *KIR2DS2* is in almost complete linkage disequilibrium (LD) with *KIR2DL2*, but is not linked to *KIR2DL3*, the functional effects attributed to KIR2DL2 could also have contributions from KIR2DS2.

**Table 3:** KIR2DL2 and KIR2DL3 interactions with HLA-C2.

KIR	Assay	Results	Reference
2DL2	Binding of KIR-Fc to HLA class I transfected cells	Direct binding to C*15:03, but not other C2	Winter et al. (1998)
2DL2 and 2DL3	Binding of KIR-Fc to bead-bound HLA	Direct binding to most C2 by 2DL2, only select ones for 2DL3newline Binding hierarchy: 2DL1 ≫ 2DL2 > 2DL3	Moesta et al. (2008)
2DL2 and 2DL3	Binding of KIR-Fc to HLA class I transfected cells	Direct binding to C*15:03	Pende et al. (2009)
2DL2 and 2DL3	Inhibition of killing by NK-92 transductants	Functional inhibition by C*04:01, C*06:01, and C*15:03newline Binding hierarchy: 2DL1 ≫ 2DL2 > 2DL3	Winter et al. (1998)
2DL2	Inhibition of killing by NKL transductants	Functional inhibition by C*04:01, and C*15:03	Moesta et al. (2008)
2DL2/3	Inhibition of killing by primary 2DL2/3^+^ NK cells	Inhibition of killing of C2^+^ leukemia blasts or C*04:01 transfectants	Pende et al. (2009)
2DL2	Inhibition of primary NK degranulation (CD107a)	Inhibition by C*04:01	Schonberg et al. (2011)

*Where 2DL2/3 is indicated, the precise KIR is not identified, where both were tested, they are listed individually.*

In addition to the subset of C1^+^ HLA-C allotypes, the HLA-B allotypes, HLA-B*46 and B*73, carry C1 and function as ligands for KIR2DL2/3 in cell-killing and direct binding assays (Barber et al., 1996; Moesta et al., 2008). Whereas all HLA-B allotypes have asparagine 80, HLA-B*46 and HLA-B*73 are the only HLA-B allotypes to combine asparagine-80 with valine 76 (Robinson et al., 2011), the latter being fixed at HLA-C and shown to be important in forming the C1 and C2 epitopes (Mandelboim et al., 1997). That neither HLA-B*46 nor HLA-B*73 is widely distributed, the former being localized to South East Asia (Abi-Rached et al., 2010) and the latter to western Asia (Abi-Rached et al., 2011) suggests these variants emerged in the human population relatively recently and underwent localized selective sweeps. One possibility is that the advantage conferred by these allotypes was their function as C1-bearing ligands for KIR2DL2/3. Whereas human C1^+^ HLA-B allotypes are rare, C1^+^ allotypes of Patr-B, the chimpanzee ortholog of HLA-B, are common (Abi-Rached et al., 2010). This contrast suggests there was much loss of C1^+^ HLA-B allotypes during human evolution, caused either by selection or genetic drift. The emergence of HLA-B*46 and HLA-B*73 can be seen as a start to reversing this long-term trend (Abi-Rached et al., 2010, 2011).

## GENETICS AND POLYMORPHISM OF KIR2DL2/3 AND KIR2DS2

**FIGURE 1:**
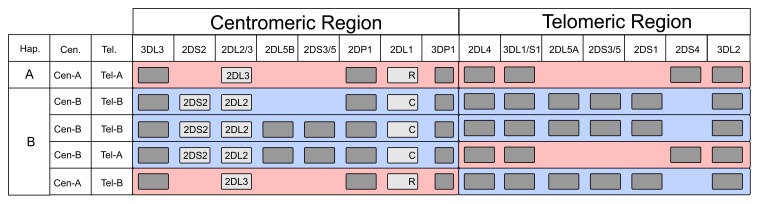
**Genomic arrangement and gene content diversity of the human KIR locus**. Gene content of common KIR haplotypes is depicted schematically: gray boxes indicate presence of the specified KIR gene; KIR2DS2, KIR2DL2, and KIR2DL3 are specifically identified to highlight the apparent allelic relationship of KIR2DL2 and KIR2DL3, and to show the linkage between KIR2DS2 and KIR2DL2. Arginine (R) and cysteine (C) residues encoded at position 245 are identified for the KIR2DL1 allotypes. “Hapl.” denotes overall haplotype designation; “Cen.” and “Tel.” denote designation of centromeric and telomeric segments, respectively. Red shading denotes A haplotype segments, blue shading denotes B haplotype segments.

KIR are encoded by a compact cluster of genes that forms part of the leukocyte receptor complex (LRC) on chromosome 19q13.4 (Wilson et al., 2000; Trowsdale, 2001). An important component of *KIR* variation is that the *KIR* haplotypes vary in gene content (Uhrberg et al., 1997; Wilson et al., 2000). Conserved genes are present at the centromeric (*KIR3DL3*) and telomeric (*KIR3DL2*) ends of the haplotype, as well as in the central part (*KIR3DP1* and *KIR2DL4*) of the locus (Pyo et al., 2010). These framework genes define two regions of gene-content variation, one in the centromeric part of the locus, the other in the telomeric part. Found in both parts of the locus are two types of alternative gene-content motifs that are qualitatively different and called *Cen-A*, * Cen-B*, *Tel-A* and *Tel-B* (**Figure 1**). The combination of *Cen-A* and *Tel-A* forms the group *A KIR* haplotype, a relatively short haplotype with a predominance of inhibitory receptors that recognize HLA class I. The three other combinations *Cen-B* with *Tel-B*, *Cen-B* with *Tel-A*, and *Cen-A* with *Tel-B* are collectively called the group *B KIR* haplotypes. Characterizing the *Cen-B* and *Tel-B* motifs are activating KIR and KIR that have reduced or lost recognition of HLA class I. Gene-content variation of *KIR* haplotypes has evolved through asymmetric recombination, which is facilitated by the short intergenic regions with high sequence similarity. Complementing this mechanism is homologous recombination at the center of the locus, which assorts the different *Cen* and *Tel* motifs (Wilson et al., 2000; Pyo et al., 2010).

*KIR2DL2/3* is a gene of the centromeric region: *KIR2DL3* being a characteristic gene of *Cen-A*, while *KIR2DL2* is a characteristic gene of *Cen-B*. A further difference is that the *KIR2DS2* gene is adjacent to the *KIR2DL2* gene and is also a characteristic gene of *Cen-B*. Indeed, *KIR2DL2* and *KIR2DS2* are in almost complete LD with each other and *KIR2DS2* has never been found adjacent to *KIR2DL3*. From a functional point of view it is important to appreciate that the combination of *KIR2DS2* and *KIR2DL2* is allelic to *KIR2DL3*, a property that complicates the interpretation of the many disease associations that correlate with the* Cen-A*/*Cen-B* difference.

Gene-content diversity of *KIR* haplotypes is one dimension to *KIR* diversity, another is allotypic polymorphism, a feature of all the HLA-C reactive KIR (**Table 4**). For KIR2DL2/3 the principal allotypic division is between KIR2DL2 and KIR2DL3, which differ at four positions in the Ig-like domains and at 10 positions in the stem, transmembrane and cytoplasmic regions. Substitutions in the Ig-like domains are responsible for giving KIR2DL2 higher avidity for C1 than KIR2DL3, and also for giving KIR2DL2 higher cross-reactivity with particular C2^+^HLA-C (notably HLA-C*02 and HLA-C*05; Moesta et al., 2008). The sequences of the stem, transmembrane, and cytoplasmic regions of KIR2DL2 and KIR2DL1 are very similar indicating that *KIR2DL2* was the product of recombination between ancestral forms of *KIR2DL3* and *KIR2DL1*. In* in vitro* assays of cellular cytotoxicity the different stem, transmembrane, and cytoplasmic regions of KIR2DL2 and KIR2DL3 had no functional effect. However, the similarity of KIR2DL2 and KIR2DL1 in these regions of signal transduction might contribute to the striking observation that the presence of the *KIR2DL2* gene is associated with reduction in the frequency of NK cells expressing KIR2DL1 (Schonberg et al., 2011).

**Table 4:** Polymorphism of HLA-C reactive KIR.

	Number of alleles	Non-functional alleles
*KIR2DL1*	24	KIR2DL1*013N
*KIR2DL2*	10
*KIR2DL3*	17	KIR2DL3*008N
*KIR2DS1*	8
*KIR2DS2*	8
*KIR2DS4*	15	KIR2DS4*003-010,*012,*13

*IPD: http://www.ebi.ac.uk/cgi-bin/ipd/*

Whereas KIR2DL2 and KIR2DL3 are divergent allotypic lineages, the polymorphism within each of these lineages is more restricted, with allotypes usually differing by one or a small cluster of substitutions. Although systematic investigation of these allotypic differences has not been performed, the study of individual allotypes illustrates a range of functional influences (**Table 5**). In the Yucpa population of South Amerindians co-evolution between C1^+^ HLA-C and KIR2DL3 is associated with a reduction in the frequency of the “old” KIR2DL3*001 allotype and its replacement by two “new” allotypes, KIR2DL3*008N and KIR2DL3*009 that differ at single nucleotide positions from KIR2DL3*001 and appear unique to the Yucpa. That KIR2DL3*008N does not make a functional protein and KIR2DL3*009 has lower avidity for C1 than KIR2DL3*001, shows that the selection changing these allotype frequencies was for reduced interaction between KIR2DL3 and C1^+^HLA-C (Gendzekhadze et al., 2009).

**Table 5:** Direct HLA interactions of KIR2DS1 and KIR2DS2.

Allele	Polymorphism	Effect	Reference
2DL2/3	P16R + R148C	Increased binding/function of 2DL2 to C1 and C2 allotypes	Moesta et al. (2008)
2DL2*004	T41R	Disruption of D1 folding leads to down regulation of cell-surface expression	VandenBussche et al. (2006)
2DL2*005	A333T	Single-nucleotide polymorphism (SNP) association with Type 1 Diabetes (T1D)	Ramos-Lopez et al. (2009)
2DL1	R245C	Transmembrane R245 increases SHP-2 binding to 2DL1*003 and 2DL1*010 leading to stronger inhibition	Bari et al. (2009)
2DL3*009	R148P	Weaker binding to C1 allotypes	Gendzekhadze et al. (2009)

KIR2DL2*004 differs from other KIR2DL2 allotypes by a cluster of three substitutions in the D1 domain. Of these, the substitution of threonine for arginine at position 41 disrupts the folding of the protein, with the result that it is retained inside the cell and does not get transported to the plasma membrane (VandenBussche et al., 2006). An example of how allelic polymorphism can directly influence signaling function is provided by *KIR2DL1*, a gene that can be present on both *Cen-A* and *Cen-B*. Each of the 25 KIR2DL1 allotypes has either cysteine or arginine at position 245 toward the end of the transmembrane domain and the beginning of the cytoplasmic domain. Arginine 245 is associated with stronger inhibitory signaling than cysteine 245 (Bari et al., 2009). That the *KIR2DL1* alleles encoding arginine 245 are predominantly on *Cen-A*, whereas the alleles encoding cysteine 245 are predominantly on *Tel-B*, illustrates the more general phenomenon that the alleles present on *Cen-A* and *Tel-A* segments are functionally different from those on *Cen-B* and *Tel-B* segments (Pyo et al., 2010). From the *KIR2DL1*, * KIR2DL2*, and *KIR2DL3* alleles described here we see how polymorphism can modulate receptor avidity and specificity for ligand, receptor integrity and cell-surface expression, and the strength of signal transduction. The study of further variants is likely to uncover aspects of HLA-C reactive KIR function that are modulated by the natural polymorphism in human populations.

## PEPTIDE INTERACTIONS FURTHER DIVERSIFY KIR–HLA INTERACTIONS

In addition to KIR interactions with the α_1_ and α_2_ domains of HLA class I, the co-crystal structures show that KIR also make direct contacts with the HLA-bound peptide, consistent with the peptide-specific differences observed in biochemical and functional studies (Malnati et al., 1995; Rajagopalan and Long, 1997; Zappacosta et al., 1997; **Table 6**). In fact, all the various KIR–HLA class I combinations examined have shown some degree of peptide selectivity (Malnati et al., 1995; Rajagopalan and Long, 1997; Zappacosta et al., 1997; Hansasuta et al., 2004; Colantonio et al., 2011).

**Table 6:** Effects of the bound peptide on the interaction of KIR with HLA-C.

KIR	HLA class I	Assay	Results	Reference
2DL2/3	C*03:04/multiple peptides	Inhibition of NK cell-mediated killing of RMA-S cells with transfected HLA class I	GAVDPLLAL or TAMDVVYAL presented on C*03:04 protect from lysis; alternative peptides showed weaker inhibition	Zappacosta et al. (1997)
2DL2 and 2DL3	C*01:02/VAPWNSLSL	Binding of KIR-Fc and inhibition of NK cell degranulation against RMA-S cells with transfected HLA class I	Screened 58 p7/p8 variants; weak versus strong (>2-fold) binding observed; binding correlated with NK activity	Fadda et al. (2010)
2DL1	C*04:01/QYDDAVYKL	Binding of KIR-Fc and inhibition of NK cell clone killing against RMA-S cells with transfected HLA class I	Substitutions at p7 and p8 (Y7E, K8E, and K8D) abrogate binding of 2DL1 to C*04:01, despite stabilizing cell-surface HLA-C	Rajagopalan and Long (1997)
2DL2	C*03:04/GAVDPLLAL	Biacore analysis	Mutation of p8 (A to Y or K) abrogates 2DL2 binding, despite stabilizing pHLA expression	Boyington et al. (2000)
2DL1, 2DL2, 2DL3, 2DS1, and 2DS2	C*04:01/QYDDAVYKL and C*03:04/GAVDPLLAL and peptide mutants	Biacore analysis	Alteration of p8 disrupts KIR/HLA binding (10X or greater); p7 contributes to affinity	Stewart et al. (2005)

KIR binding and function are particularly sensitive to the residue at position 8, with the neighboring position 7 contributing an additional, but weaker effect (Rajagopalan and Long, 1997; Boyington et al., 2000). These findings are compatible with the footprints of bound KIR2DL1 and KIR2DL2 on the HLA-C*04:01 and HLA-C*03:04 molecules, respectively, that show interaction with residues 7 and 8 of the peptide (Boyington et al., 2000; Fan et al., 2001). For KIR2DL2 interaction with C1, glutamine 71 of KIR forms a hydrogen bond with the amide nitrogen of the alanine at position 8 of the peptide. Additional KIR residues (lysine-44, serine 184, and asparagine-187), which are in close proximity to the peptide bound by HLA-C, further restrict the size of the residue at position 8 in the peptides that permit KIR binding. This limitation favors small residues such as alanine and serine, while disfavoring large side chains (Boyington et al., 2000; Fan et al., 2001). The crystal structure of KIR2DS2 revealed a displacement of glutamine 71 that is predicted to prevent hydrogen bonding with the main chain nitrogen of peptide residue 8, which likely contributes to the poor C1 binding observed for KIR2DS2 (Saulquin et al., 2003).

Moreover, peptide-HLA combinations that bind KIR weakly appear to function as peptide antagonists, by competing with strongly binding peptides for complex formation with HLA-C. Fadda et al. (2010) identified peptides that bind to and stabilize the cell-surface expression of HLA, but do not support a high affinity KIR/HLA interaction. These peptides compete with KIR-permissible peptides for availability of HLA-C, and downmodulate KIR-mediated inhibition by lowering the number of inhibitory KIR ligands on target cells. Such sensitivity to peptide interactions could enable NK cells to sense subtle changes in the peptide repertoire, as occurs during viral infections. In principle this would be a more sensitive sensory mechanism than the large-scale alterations of HLA expression on target cells that cause self-HLA class I to be missing (Rajagopalan and Long, 2010). Fadda et al. (2010) also found that this effect is more prevalent for KIR2DL3 than for KIR2DL2, presumably due to the stronger inhibitory interaction of the later with C1.

## CO-EVOLUTION OF MHC-C WITH LINEAGE III KIR

Comparison of KIR specificities in human and non-human primate species shows has that KIR recognition of MHC-A and MHC-B preceded the emergence of MHC-C and its evolution to become a more specialized and superior KIR ligand than MHC-A and MHC-B (Older Aguilar et al., 2011). Co-evolving with MHC-C are the lineage III KIR, that includes all the MHC-C reactive KIR and which were expanded from a single gene into a family of genes with the emergence of MHC-C. When MHC-C first evolved from an MHC-B-like gene, in the common hominid ancestor, it carried only the C1 epitope, a state preserved by modern orangutan MHC-C. Through mutations at position 80 in MHC-C and position 44 in lineage III KIR, C2 (lysine-80) evolved from C1 (asparagine 80), and C2-specific KIR (methionine 44) evolved from C1-specific KIR (lysine-44). This involved an intermediate form of KIR that reacted with both C1 and C2 and had glutamate 44 (Moesta et al., 2009; Older Aguilar et al., 2010). In its reactivity with C1 and cross-reactivity with C2, KIR2DL2 has similarity to this intermediate.

One characteristic feature of the co-evolution between variable NK cell receptors and MHC class I ligands is the transience of individual ligand–receptor pairs, and even whole systems of ligand–receptor pairs. A second feature is formation of inhibitory and activating receptors with similar specificities for MHC class I. In such pairs of receptors the inhibitory receptors tend to be longer lasting, whereas their activating counterparts tend to become subject to selection that attenuates their function (Abi-Rached and Parham, 2005). This trend is most apparent in the human species, and exemplified by KIR2DS2, for which C1 binding is undetectable. In contrast, chimpanzee, gorilla, and orangutan all have activating C1-specific KIR for which the binding to C1 bearing MHC-C is readily detected (Moesta et al., 2010).

## EDUCATION, REPERTOIRE, AND VARIEGATED EXPRESSION

Although much debated, almost all mature human NK cells express at least one inhibitory receptor that recognizes a ubiquitously expressed self-HLA class I molecule: either HLA-A, B, C, or E (Valiante et al., 1997). Because KIR and HLA are not genetically linked on the same chromosome, the co-evolution of KIR with HLA class I cannot lead to the co-segregation in human populations of favorable combinations of KIR and HLA class I. This property combines with the polymorphism of both receptors and ligands, to produce a situation in which numerous individuals have KIR without a cognate HLA class I ligand and/or an HLA ligand without its cognate KIR.

The HLA class I receptors are expressed at late stages in human NK cell development, with CD94:NKG2A, being expressed first (Miller and McCullar, 2001; Freud et al., 2006; Cooley et al., 2007). CD94:NKG2A provides the NK cell with a guaranteed inhibitory receptor for self-HLA class I. Subsequently, transcription is initiated at the *KIR* locus. Here, a system of competing sense and anti-sense promoters (Davies et al., 2007; Stulberg et al., 2007) means that each NK cell expresses only a subset of the *KIR* genes, a phenomenon described as variegated expression (Trowsdale, 2001), which in turn imparts considerable phenotypic diversity to the NK cell population. Once an NK cell expresses KIR they can survey the set of self-MHC class I to see if it includes a cognate ligand. If there is no such ligand, then the NK cell uses CD94:NKG2A as its self receptor (Grzywacz et al., 2006). If a KIR engages its cognate ligand then expression of CD94:NKG2A is turned off and the NK cell uses the KIR as its self receptor (Yu et al., 2010). This process, whereby a developing NK cell is influenced by the signals generated through engagement of ligand by a self-MHC class I receptor, is called NK cell education (Anfossi et al., 2006).

In addition to the C1 and C2 epitopes recognized by lineage III KIR, two other HLA epitopes are recognized by the lineage II KIR. These comprise the Bw4 epitope carried by subsets of HLA-A and -B allotypes, and the A3/11 epitope carried by a small minority of HLA-A allotypes. Of the four epitopes C1, C2, and Bw4 can educate NK cells, but the A3/11 epitope cannot (Fauriat et al., 2008; Yawata et al., 2008). With few exceptions every human individual has either C1 or the C2 epitope (or both) to educate NK cells, whereas 25% of the human population lack the Bw4 epitope (Norman et al., 2007). This again illustrates the leading role played by HLA-C in providing KIR ligands.

While the mechanisms underlying NK cell education are poorly understood and remain a matter for debate, there is good evidence showing that HLA-C reactive KIR play a role in education (Anfossi et al., 2006; Fauriat et al., 2008, 2010; Yawata et al., 2008). What is less certain is the extent to which the selective process of NK cell education, which varies with HLA class I type, influences the NK cell repertoire of KIR expression. Investigation of this question by several groups has led to different conclusions. Study of Japanese donors (most of whom were homozygous for *A KIR *haplotypes) reported a strong influence of HLA-C type on the distribution of inhibitory KIR (Yawata et al., 2008), whereas a study of European donors who were also homozygous for* A KIR *haplotypes found no correlation between HLA-C allotype distribution and KIR expression (Andersson et al., 2009). But another study of European donors homozygous for* A KIR *haplotypes detected a dominant effects of C2 or C1 homozygosity on the frequencies of KIR2DL1 and KIR2DL3/3 expression by NK cells (Schonberg et al., 2011). Thus, in C2 homozygous donors, NK cells expressing KIR2DL1 were most frequent, whereas in C1 homozygous donors NK cells expressing KIR2DL2 were most frequent. In donors with a *B KIR* haplotype, the effect of C2 on the frequency of KIR2DL1 expression was abrogated by the presence of *KIR2DL2*.

KIR2DS1 also contributes to NK cell education. KIR2DS1 expressing NK cells are hyporesponsive to C2/C2 targets, even if those NK cells expressed KIR2DL3 or CD94/NKG2A (Fauriat et al., 2010). This is consistent with the ability of KIR2DS1 to override NKG2A-mediated inhibition on NK cells educated in the absence of C2, but subsequently exposed to C2-bearing target cells, as would occur during haploidentical bone marrow transplantation (Foley et al., 2008).

## FUNCTION: ROLE IN INFECTION

The heightened susceptibility of patients with NK cell deficiencies to recurrent viral infections (Orange, 2002) strongly implicates NK cells in protection against viral infections. Subsequent epidemiological studies have correlated either KIR alone, or combinations of KIR and HLA class I with susceptibility, resistance, and chronicity of viral infections (Khakoo and Carrington, 2006).

Increased resolution of acute hepatitis C viral infection was observed for individuals who were homozygous for KIR2DL3 and HLA-C1. This report concluded that the weaker inhibitory interaction of KIR2DL3 with C1, but not the stronger inhibitory interaction of KIR2DL2 and KIR2DL1 with C1 and C2, respectively, allows penetrance of activating signals during viral infection (Khakoo et al., 2004).

Similar relationships of activation versus inhibition have been seen in studies on the recurrence of cytomegalovirus (CMV) infection following hematopoietic cell transplantation (HCT), where a larger number of activating KIR correlates with fewer recurrences (Chen et al., 2006; Zaia et al., 2009). Similarly, recurrence of CMV infection following kidney transplant appears to be reduced in the presence of multiple activating receptors (Stern et al., 2008) and/or in the absence of inhibitory KIR–ligand pairs (Hadaya et al., 2008). Specifically, absence of either interaction between KIR2DL1 and C2, or between KIR2DL2/3 and C1 were found to be the primary components of this association (Hadaya et al., 2008).

For HIV infection an extensive set of associations has been made with the Bw4 epitopes carried by HLA-B and the KIR3DL1/S1 receptors of lineage II (Martin et al., 2002, 2007; Alter et al., 2007). More recently, evidence for interplay between HIV and KIR2DL2 has been reported (Alter et al., 2011). In individuals who have KIR2DL2 and are HIV-infected, KIR2DL2^+^ NK cells are activated by virus-infected CD4 T cells. This interaction appears to select for variant viruses, in which the capacity of KIR2DL2 to bind and/or respond to infected CD4 T cells is lost. The implication of this finding is that variant viral peptides alter the repertoire of peptides bound by HLA-C, causing NK cell inhibition instead of an NK cell response. Although the authors focus on KIR2DL2, they cannot rule out involvement of KIR2DS2 (Alter et al., 2011). Furthermore, individuals with HLA-C allotypes that have genetically determined high cell-surface expression, progress more slowly to AIDS and control the viral load significantly better than individuals with low HLA-C expressing alleles (Thomas et al., 2009). Unknown, however, is whether this effect is directly related to KIR/HLA-C interactions or presentation to cytotoxic T cells, since no correlation has been found with specific HLA-C reactive KIR.

For two viral infections with known HLA class I associations, human T lymphotropic virus type 1 (HTLV-1) and HCV, the presence of KIR2DL2 was correlated with enhanced protection when combined with protective HLA class I (HLA-C*08 for HTLV-1 and HLA-B*57 for HCV), but exacerbated the detrimental effect of HLA-B*54 on HTLV-1 (Seich Al Basatena et al., 2011). Rather than mediating a direct NK cell-mediated effect, it is proposed that KIR2DL2 expressed on CD8^+^ T cells modulates the T cell response to the virus (Seich Al Basatena et al., 2011). Similar effects of KIR on CD8^+^ T cell responses have been reported in the context of HIV (Alter et al., 2008), EBV (Poon et al., 2005), and CMV (Chen et al., 2006; van der Veken et al., 2009).

## FUNCTION: ROLE IN AUTOIMMUNITY

Susceptibility to numerous autoimmune diseases has been correlated with genes in the HLA complex, and frequently they are the strongest genetic associations with the disease (Lechler and Warrens, 2000). With some notable exceptions, such as HLA-B*27 with ankylosing spondylitis and psoriasis with HLA-C*06 (Diaz-Pena et al., 2009; Reveille, 2011), the stronger associations have tended to be with the HLA class II genes. Because HLA class I and KIR form functional ligand–receptor pairs there is the appealing possibility that combinations of particular functionally interacting HLA and KIR variants will give stronger correlations with disease than either component alone.

Many exploratory studies have been made on cohorts of patients and controls that were previously analyzed for HLA type, and subsequently typed for* KIR* gene-content diversity. Some examples of the results are shown in **Table 7**. The foundation for human KIR diversity is the difference between the *A *and *B *haplotypes and their constituent centromeric and telomeric gene motifs. A common feature of the associations with autoimmune diseases is that *B *haplotypes, or their components, are associated with susceptibility to autoimmune disease (Parham, 2005). For example, susceptibility to psoriasis vulgaris is correlated with the presence of KIR2DS1 and HLA-Cw*06 (Luszczek et al., 2004; Suzuki et al., 2004), whereas the risk for type I diabetes risk is elevated for individuals having the receptor–ligand combination of KIR2DS2 and HLA-C1, but lacking the C2 and Bw4 epitopes that engage other inhibitory KIR (van der Slik et al., 2003). In other instances, the contribution of individual KIR is more difficult to establish.

**Table 7:** Associations of KIR2DL2/3 with autoimmune disease.

	Disease associated and HLA factors	Reference
**KIR associations**
Psoriatic arthritis	Presence of *2DS1 *is associated with disease	Williams et al. (2005)
Scleroderma	Presence of *2DS2*, absence of* 2DL2* predisposes to disease	Momot et al. (2004)
Scleroderma	Presence of *2DS1* and/or *2DS2* is increased in diseased individuals	Pellett et al. (2007)
Type 1 diabetes	Presence of *2DL2* and *2DS2* positively correlates with disease	Nikitina-Zake et al. (2004)
Type 1 diabetes	SNP coding for A333T polymorphism in *2DL2* is associated with disease	Ramos-Lopez et al. (2009)
Rheumatoid arthritis	Increased frequency of 2DS2 expression on NK cells and T cells in patients that develop vasculitis; relevant expression is thought to be on CD4^+^CD28^-^ T cells	Yen et al. (2001)
Systemic lupus erythematosus	Frequency of *2DL2* and *2DS1* are increased in SLE patients	Hou et al. (2010)
Systemic lupus erythematosus	Presence of *2DS1 *with absence of *2DS2* is associated with disease	Pellett et al. (2007)
**KIR + HLA associations**
Crohn’s disease	*2DL2/3 *heterozygosity + C2 homozygosity is protective; *2DL2/3 *heterozygosity + presence of C1 is predisposing	Hollenbach et al. (2009)
Ulcerative colitis	*2DL2/2DS2* are overrepresented in patients; *2DL3* in the presence of C1 is protective	Jones et al. (2006)
Psoriatic arthritis	Susceptibility determined by combinations of /HLA combinations; absence of inhibitory HLA ligands is predisposing	Nelson et al. (2004)
Psoriasis vulgaris	*2DS1 *and *KIRB* haplotypes are correlated with disease	Suzuki et al. (2004) Luszczek et al. (2004)
Type 1 diabetes	*2DS2* and C1 predisposes to disease; *2DL1 *and C2 is protective	van der Slik et al. (2003)
Type 1 diabetes	Combination of *2DL2* and C2 confers susceptibility, absence of *2DL2* and C2 is protective. Either effect is stronger in the absence of *2DS1 and 2DS2*	Shastry et al. (2008)
Sjogren’s syndrome	Presence of *2DS2*, in absence of* 2DL2* predisposes to disease. Effect is strongest when C1 is present	Lowe et al. (2009)
Multiple sclerosis	*2DS1 *is protective; effect is stronger in the presence of C2	Fusco et al. (2010)

As we have emphasized here, the formidable linkage disequilibrium between KIR2DL2 and KIR2DS2 makes it difficult to distinguish their respective contributions, as in ulcerative colitis. Here KIR2DL3 in the presence of HLA-C1 had a protective effect, while the presence of KIR2DL2/2DS2 increased the risk of disease (Jones et al., 2006). Similarly, *B* haplotype *KIR2DL2*, but not A haplotype *KIR2DL3* has been linked with several autoimmune conditions, including type I diabetes (van der Slik et al., 2003), psoriatic arthritis (Martin et al., 2002; Nelson et al., 2004), and ulcerative colitis (Jones et al., 2006). In dissecting the separate roles of KIR2DS2 and KIR2DL2, rare *KIR* haplotypes that have the *KIR2DS2* gene without the neighboring *KIR2DL2* gene are likely to be informative. In studying scleroderma patients, Momot et al. (2004) showed that disease was associated with the individuals who had *KIR2DS2* but lacked *KIR2DL2*. At face value it seems that an upset in the usual balance between KIR2DS2 and KIR2DL2 is the cause of the increased risk of disease.

On several counts the interpretation of the disease associations has not been simple. First, the data are often not sufficiently robust, because of the small sizes of the cohorts examined and the lack of replication in a further cohort. Second, the associations often involve KIR for which functions and ligands are poorly understood, as exemplified by KIR2DS2. Thirdly, because KIR are expressed both on NK cells and T cells of memory phenotype (van Bergen and Koning, 2010), the cellular basis for the genetic correlations is not established. Lastly, because the disease-association studies are usually restricted to examining the presence and absence of *KIR *genes they are insensitive to the rich allelic polymorphism in which the *KIR* factors associated with autoimmunity or more likely to be found.

## FUNCTION: ROLE IN HEMATOPOIETIC CELL TRANSPLANTATION

Hematopoietic cell transplantation has been to the fore of research on KIR, because of observations made on leukemia patients who received an HLA haploidentical transplant from a relative when no HLA-identical donor was available from either the family or the international registries of unrelated donors (Ruggeri et al., 2002). For these transplants the shared HLA haplotype enables the donor-derived lymphocytes to interact with the HLA class I and II antigens expressed by the recipient’s non-hematopoietic cells. The mismatched HLA haplotype can cause a burst of alloreactive NK cells in the transplanted patient that can improve the outcome by reducing the likelihood of leukemic relapse, presumably by killing residual leukemia cells. Such a graft-versus-leukemia (GVL) effect is principally seen against myeloid leukemias and not lymphocytic leukemias, perhaps reflecting the physiological interactions that occur between NK cells and myeloid cells, notably dendritic cells.

The occurrence of the GVL effect is determined by the HLA mismatch between the donor and recipient, particularly in the distribution of the C1 and C2 epitopes. A GVL effect occurs when the donor has an epitope, either C1 or C2, that the recipient lacks. This is a case of missing-self recognition: some NK cells that have become educated on the donor’s HLA-C allotypes are unable to be inhibited by the recipient’s cells including the leukemia. The burst of alloreactive NK cells appears transient, for as the patients hematopoietic system becomes fully reconstituted a state of tolerance is reached. The GVL can also provide the recovering transplant recipient protection from CMV infection (Velardi et al., 2012).

As in the case of the autoimmune diseases, many of the studies rely on cohorts of transplant donor and recipient pairs, for which HLA-C types and clinical outcomes were reanalyzed in the context of the C1 and C2 epitopes. The implied effects of alloreactive NK cells on transplant outcome are mixed, and appear to depend on differences in the protocols used for transplantation. A key feature of the transplants described by Velardi and colleagues (Ruggeri et al., 2002; Velardi et al., 2012) is rigorous depletion of T cells from the graft. In contrast, in transplants performed with T cell replete grafts a detrimental effect was found when the donor has an epitope lacking in the recipient (Sun et al., 2007).

*KIR* genes also influence the outcome of HCT, but also only for recipients treated for myeloid leukemia. Donors with *KIR B* haplotypes give better outcome than donors who are *A *haplotype homozygotes (Cooley et al., 2009). This effect is mainly due to the *Cen-B *motif, but there is also a contribution from *Tel B* (Cooley et al., 2010). Thus it is the combination of *KIR2DL2 *and *KIR2DS2* in the donor that is implicated in the effect, which is also improved by the recipient having the C1 epitope, the main ligand for KIR2DL2 (Cooley et al., submitted).

## FUNCTION: ROLE IN PREGNANCY OUTCOME

A subset of NK cells resident in the uterus plays a critical role in formation of the placenta early in pregnancy. By modulating the invasion of fetal extravillous trophoblast cells (EVTs), uterine NK (uNK) cells affect the remodeling of maternal spiral arteries, a process that is crucial for ensuring sufficient blood and nutrient flow across the placenta. Insufficient trophoblast invasion has been implicated in pregnancy disorders, including preeclampsia, recurrent miscarriage, and fetal growth restriction. KIR interaction with HLA-C appears to play a major role in controlling this interaction. This is because EVT express HLA-C but not HLA-A or HLA-B, thus HLA-C is the only polymorphic HLA class I expressed by the fetal cells that contact the maternal circulation (Apps et al., 2009). Correspondingly, the KIR repertoires of uNK cells are skewed toward HLA-C reactive KIR and express these receptors at high levels on the cell surface, as evidenced by increased staining with HLA-C tetramers (Sharkey et al., 2008). Paralleling the situation in HCT, the independent genetic segregation of KIR and HLA provides for the potential in pregnancy to have mismatches between maternal KIR-bearing NK cells and fetal HLA class I, since EVT express both the maternally and paternally inherited *HLA-C* alleles (Hiby et al., 2010).

Epidemiological studies have shown an increased risk for preeclampsia, recurrent miscarriage, and fetal growth restriction for mothers who are homozygous for the *KIR A* haplotype, an effect that is further increased when the fetus expresses the C2 epitope, particularly if inherited from the father (Hiby et al., 2004, 2010). Thus interaction of inhibitory KIR2DL1 on maternal uNK cells with C2 on EVT is implicated in increasing the risk of pregnancy disorder. A protective effect is correlated with *KIR2DS1 *on *Tel-B*, and is presumably caused by functional interaction of this activating NK cell receptor with C2, suggesting that the increased activation of maternal uNK cells stimulated greater trophoblast invasion (Hiby et al., 2010). Furthermore, paternal inheritance of C2 appears to play a dominant effect, suggesting that education of uNK cells in the presence of maternal C2 may dampen the influence of an unfavorable KIR–HLA combination.

None of the C1-reactive KIR have been directly implicated in affecting susceptibility to pregnancy disorders, but have to be considered in light of the allotypic relationship of C1 with C2. Hiby et al. (2004) point to a pronounced inverse correlation between frequencies of C2 and KIR *A* haplotype across world populations, as evidence for the powerful selective pressure exerted by factors influencing reproductive success.

## CONCLUDING REMARKS

Prompted by observations of HSCT (Ruggeri et al., 2002), responses to viral infections (Khakoo et al., 2004), and success in pregnancy (Hiby et al., 2004), it is now appreciated that HLA-C reactive KIR play a critical role in NK cell function, but must be examined in the context of their ligands. Beyond the allotypic delineation between C1 and C2, understanding how HLA polymorphism affects NK cell function is limited. Owing to the highly polymorphic nature of the *HLA-C* locus, allelic effects on peptide repertoire, cell-surface expression and on the KIR–HLA interface are all likely to further diversify KIR–HLA interactions, but they have yet to be studied in depth. Similarly, allelic differences of the HLA-C reactive KIR have been studied for their effects on the interaction with HLA-C. A recent in-depth analysis of positively selected residues of KIR2DL1 and KIR2DL3 revealed subtle modulation of the KIR–HLA interaction (Hilton et al., 2012), and also suggests that HLA-C*04:01, a C2 allele commonly used in functional and biochemical assays may have properties that are not representative of all C2 allotypes. These findings strengthen the need to further our understanding of the diversified interactions of KIR with HLA-C.

## Conflict of Interest Statement

The authors declare that the research was conducted in the absence of any commercial or financial relationships that could be construed as a potential conflict of interest.
